# The In Vivo Effect of Water-Based Lubricants on the Vaginal Microbiome of Women from Varying Age Groups: Exploratory Analysis of a Randomized Controlled Trial

**DOI:** 10.3390/microorganisms12091917

**Published:** 2024-09-20

**Authors:** Jose A. Freixas-Coutin, Jin Seo, Sarah Hood, Michael Krychman, Santiago Palacios

**Affiliations:** 1 Reckitt Health US LLC, 1 Philips Pkwy, Montvale, NJ 07645, USA; jin.seo@reckitt.com; 2 Reckitt Benckiser Healthcare Ltd., Hull HU8 7DS, UK; sarah.hood@reckitt.com; 3 The Southern California Center for Sexual Health and Survivorship Medicine, Newport Beach, CA 92663, USA; mkrychman@me.com; 4 Palacios Institute of Health and Women’s Medicine, 28009 Madrid, Spain; spalacios@institutopalacios.com

**Keywords:** vaginal microbiome, (personal) lubricants, pH, osmolality, vaginal dryness, menopause

## Abstract

Vaginal mucosa undergoes physiological changes across the lifespan, such as increased pH and reduced natural lubrication which are known to impact vaginal commensal microorganisms, hence increasing the chances of vaginal infections. An improved understanding of vaginal microbiome composition in different age groups and the effect of social behaviors, such as the use of personal lubricants, could facilitate the development of new strategies to maintain good vaginal health. The objective of this study was to assess the effect of water-based lubricants on the human vaginal microbiome. Fifty females from three age groups (18–29, 30–44, and 45–65 years) with mild-to-moderate vaginal dryness were randomized to one of five lubricants (four of which were formulated to meet expert guidance on osmolality and pH). Subjects entered the study at tolerance or treatment phase (vaginal intercourse minimum once a week using assigned lubricant). Four vaginal swabs per participant were sampled during pre-(“baseline”), post-first (“2 h”, “24 h”) and post-last (“4 weeks”) lubricant application to assess bacterial and fungal diversity via amplicon sequencing. Vaginal pH and relative humidity were measured at baseline, 2 h, and 24 h post-lubricant application. Relative bacteriome abundance was statistically compared between timepoints for each lubricant group. Vaginal moisture, age, BMI, and pH were correlated with bacteriome relative abundance. Lactobacilli and *Gardnerella* sp. Were predominant across participants. Repeated lubricant application did not significantly alter the vaginal bacteriome during 4 weeks of product use (*p* > 0.05) when measured by relative abundance and alpha-diversity index. Bacteriome diversity and abundance differed significantly between age groups at baseline whereas lactobacilli relative abundance was negatively associated with age and vaginal pH.

## 1. Introduction

The human vaginal microbiome is comprised of microbial genetic profiles coexisting in a mutualistic relationship with the host. They may include bacteria, fungi, and viruses with major implications in health and disease [1]. Vaginal commensal bacteria represent the first line of defense by preventing colonization of opportunistic pathogens [2]. Lactobacilli appear to be the most prevalent species in healthy women of reproductive age. They produce lactic acid from glycogen released by the vaginal epithelium, lowering vaginal pH and inhibiting growth of opportunistic pathogens [2,3,4].

During the onset of menopause, estrogen levels and glycogen secretion decrease [5,6]. This is known to reduce lactobacilli, while enabling colonization of facultative and obligate anaerobic, and enteric bacteria. This microbial shift, which also causes an increase in vaginal pH, may enable colonization of opportunistic microbes such as *Escherichia coli*, *Candida* spp., *Enterobacter* spp., and *Gardnerella* spp. [3,6]. Hormonal and microbial changes can have a profound effect on the gynecological health of menopausal women. Such alterations are associated with genitourinary symptoms (i.e., vulvovaginal atrophy and vaginal dryness) which can reduce quality of life [7]. Non-hormonal remedies such as the use of over the counter (OTC) vaginal lubricants are generally recommended as a first-line treatment to alleviate vaginal dryness [8,9,10]. However, some commercial lubricants contain ingredients and additives (e.g., chlorhexidine), suggesting potential negative consequences, including vaginal endothelial irritation [8] and disruption to the microbiome [11]. Additionally, some personal lubricants may disturb the delicate balance of the vaginal pH [12] which is essential for an optimal vaginal microbiome. There is an increasing understanding around formulating personal lubricants to be as “body friendly” as possible, as demonstrated by the World Health Organization (WHO) guidance issued on osmolality [13]. Ideally, personal lubricants should ease vaginal dryness without disturbing vaginal physiology, pH, or microbial communities.

Limited studies have only tested the in vitro efficacy of personal lubricants to maintain the vaginal microbiome. One major finding has been the inhibition of *Lactobacillus crispatus* growth upon in vitro exposure to a selection of OTC vaginal products [11,14,15]. Lactobacilli, including *L. crispatus*, *L. gasseri*, and *L. helveticus*, help maintain a protective microbial barrier in the vagina of reproductive-age women [2,16]. Therefore, understanding the in vivo impact of personal lubricants on the vaginal microbiome is an important, yet understudied parameter. The present study evaluated the effect of five water-based personal lubricants on vaginal microbiome communities in females of different age groups. Vaginal pH and relative humidity were measured to determine any potential changes derived from lubricant application.

## 2. Materials & Methods

### 2.1. Study Subjects and Clinical Design

This open label, five arm, parallel-design study was conducted from 1 March 2021 to 29 June 2021 by proDERM GmbH (Hamburg, Germany). A total of 183 female subjects aged 18–65 years were recruited for the study. Eligible participants were randomized in a 1:1:1:1:1 ratio to one of five lubricants using permuted blocks of fixed size. Randomization was stratified by menopausal status in a 1:1 ratio of pre- and post-menopausal status. Supplementary information on subject demographics is provided in Supplementary Tables S1 and S2. Blinding procedures were not implemented as this was an open label, parallel-design study, with no intention to compare between lubricants. Written informed consent was granted from all subjects prior to enrollment. Inclusion criteria were as follows: monogamous heterosexual relationship, use of highly effective contraception methods, mild to moderate vaginal dryness and dyspareunia, and no other significant disease in the test region (vagina and vulva) as indicated by a gynecologist. Additional post-menopausal inclusion criteria included the absence of menstrual cycles for at least 12 months due to natural or premature events (i.e., surgical and chemotherapy). Subjects who were pregnant, breast-feeding, trying to conceive, had reported allergic reactions to personal lubricants and their ingredients, or showed any sign of urinary or vaginal infection (including sexually transmitted infections), were excluded from the study. Moreover, clinical site visits for pre-menopausal subjects were arranged in accordance with the subject’s menstrual cycle, to avoid visits occurring during menstrual bleeding as changes to hormone levels during menstruation can impact glycogen availability, pH, and the overall vaginal microbiome [5,6]. Additional exclusion criteria along with the complete clinical trial protocol can be retrieved from ClinicalTrials.gov with the identifier NCT04908124. The primary results (including CONSORT diagram) have been published in full [17].

The clinical study consisted of two phases; a tolerance phase followed by a treatment phase. The majority of subjects were enrolled directly to the treatment phase which assessed Female Sexual Function Index as the primary endpoint [17]. In the present manuscript, we report on the subset of 50 eligible subjects who were selected to enroll first to the tolerance phase to measure vaginal pH, relative humidity, and microbiome profiles as exploratory endpoints. Subjects were randomized to one of five treatment groups (approximately 9–11 subjects per group), and stratified by three age groups (18–29, 30–44, and 45–65 years). Treatment groups consisted of five water-based personal lubricants (herein referred to as lubricants A to E) from two brands (Durex and KY; Reckitt, Slough, UK). All were suitable for vaginal, oral, and anal sexual intercourse. While iso-osmolar lubricants may be considered optimal [18], lubricants B, C, D, and E had been formulated to comply with interim WHO guidance which states osmolality should be ≤1200 mOsm/kg and have an acidic pH, aligned to a healthy vaginal range (3.8–4.5) [13]. Lubricant A was a hyperosmolar product from the existing portfolio that did not meet the WHO osmolality guidance. It was included to account for any potential effect of reduced lubricant osmolality across all study timepoints. Further information on lubricant osmolality and pH is available in Supplementary Table S3.

During the tolerance phase, a 3 day wash out period (herein defined as baseline) was followed by two clinical site visits. Subjects were instructed to apply a single application (approx. 3 g) of their allocated lubricant. Clinical assessments were carried out at baseline, 2 h, and 24 h post-lubricant application. Over the 4-week treatment phase after the tolerance phase, subjects were asked to apply their assigned lubricant when engaging in sexual intercourse at least once a week. Adverse events (AEs) were recorded in subject diaries and captured during scheduled visits via non-leading questions. AEs were classified by severity, as either mild (slight discomfort, not limiting everyday activities), moderate (sufficient discomfort, interfering with everyday activities), or severe (intolerable discomfort, preventing everyday activities). During the study, no serious adverse effects were observed and 78 non-serious adverse effects were reported (of which 74 were mild and 4 were of moderate severity). Further methodological information on this clinical study has been published elsewhere in full [17].

### 2.2. Study Assessments and Endpoints

During the tolerance phase, a thermo-hygrometer device equipped with a vaginal sterile probe (Tecnolab del Lago Maggiore S.r.l., Verbania, Italy) was used to measure the vaginal moisture at baseline, and at 2 and 24 h post-lubricant application. Vaginal moisture was measured in triplicate for each timepoint and expressed as percentage change in relative humidity. Additionally, vaginal pH was measured during the tolerance phase using ColorpHast™ pH strips of 4.0–7.0 pH range (Thermo Scientific, Waltham, MA, USA) at baseline, and at 2 and 24 h post-lubricant application. For microbiome analysis, a total of 197 vaginal swabs were sampled in duplicate using the DNA/RNA Shield Collection Tube with swab kit (R1109, Zymo Research, Irvine, CA, USA), from the walls of the vagina, approximately 3–5 cm in from the external vaginal orifice towards the lateral fornix, ensuring the sterile swabs were rotated for full coverage. Vaginal swabs were sampled in duplicate and pooled to ensure high gDNA yield. Samples were collected during the tolerance phase at pre-(“baseline”) and post-first (“2 h” and “24 h”), and during the treatment phase at post-last (“4 week-visit”) lubricant application. Three subjects did not complete the study due to unmet eligibility criteria or failure to attend the 4-week visit, hence their 4-week swabs were not collected for microbiome analysis. All samples and measurements were taken by a certified gynecologist. Additional subject data collected during the study included age, height, weight, body mass index (BMI), and race.

### 2.3. Microbiome Analysis

Following sampling, vaginal swabs were stored in DNA/RNA Shield stabilizing transport medium (Zymo Research, Irvine, CA, USA) at −20 °C until required for DNA extraction. Total DNA was extracted and prepared for next generation amplicon sequencing using BaseClear standard protocols (Leiden, The Netherlands). The V3-V4 hypervariable 16S and ITS1 rRNA gene regions (herein referred to as 16S and ITS1) represented the barcodes for bacterial and fungal identification, respectively. It is worth noting that sequencing of the ITS1 region was only performed in 13 DNA samples as the remaining 184 samples were negative for fungal DNA.

Briefly, DNA samples were PCR amplified using the Phusion HF PCR Master Mix (ThermoFisher Scientific, Waltham, MA, USA) and the following 341F (5′-cctacgggnggcwgcag-3′) and 785R (5′-gactachvgggtatctaatcc-3′) primers for the 16S region, and a pool of ITS1-specific primers optimized by Bellemain et al. (2010) for fungal barcoding studies [19]. Amplicon libraries were prepared for sequencing on the Illumina MiSeq instrument (Illumina, San Diego, CA, USA) at 301 × 2 cycles.

All 16S- and ITS1-specific primers were synthesized along with the Illumina overhang adapters attached to the 5′ region. The PCR program consisted of an initial denaturation step at 95 °C for 3 min, followed by 40 cycles consisting of denaturation at 95 °C for 30 s, annealing at 55 °C for 30 s, elongation at 72 °C for 30 s, and a final extension step at 72 °C for 5 min in an Applied Biosystems QuantStudio^TM^ 5 Real-Time PCR system (ThermoFisher Scientific, Waltham, MA, USA). PCR amplicons were ligated to nextera DNA combinatorial dual (CD) indexes using the Phusion HF PCR Master Mix following the same PCR conditions with a reduced number of cycles. The indexed amplicon libraries were quantified, and quality checked via fluorescence-based methods and capillary electrophoresis systems. Amplicon libraries were denatured, normalized, and multiplexed following Illumina recommended protocols, and loaded onto an Illumina MiSeq instrument (Illumina, San Diego, CA, USA) for paired-end sequencing at 301 × 2 cycles. The sequences generated with the MiSeq instrument were performed under the scope of accreditation for BaseClear B.V. (L457; NEN-EN-ISO/IEC 17025).

The FASTQ read sequence files were generated using “Bcl2fastq” package version 2.20 (Illumina, San Diego, CA, USA). The initial quality assessment was based on data passing the Illumina chastity filtering in the MiSeq Reporter Software version 2.6. Thereafter, reads containing PhiX control signal were removed using a BaseClear filtering protocol, and demultiplexed FASTQ files were used for downstream analyses. All bioinformatics and statistical analyses described henceforth were performed in RStudio v.4.3.0 [20]. Non-biological reads corresponding to Illumina CD indexes as well as 16S- and ITS1-amplification primers were trimmed from the raw reads using the “Cutadapt” tool version 4.0. Trimmed reads were fed into the “Dada2” package version 1.28 for the subsequent steps. Reads with a Phred score lower than 10 were filtered out while keeping a minimum length of 100 bases. Forward and reverse filtered reads were merged allowing a minimum overlap of 21 bases and a maximum of 3 mismatching bases to eliminate sequencing artifacts and chimeric reads (Supplementary Table S4). The resulting non-chimeric and cleaned reads were converted to amplicon sequence variants (ASVs) and classified using the Silva high quality rRNA gene database version 138 for bacteria [21] and the UNITE database general FASTA release version 10.05.2021 for fungi [22]. After taxonomic assignment, ASVs from unclassified phylum and class ranks, as well as those classified within the mitochondria family, were removed from the analysis.

Classified ASVs and their respective Operational Taxonomic Unit (OTU) counts were merged with the clinical study meta-data using the package “Phyloseq” version 1.46. The agglomeration function *tax_glom()* was used to merge OTUs with the same taxonomic classification in each sample at the genus and species levels. Taxa present 3 times or less in at least 20% of the samples were removed from the analysis as these are taxa with small abundance means and large coefficients of variation. OTU counts were normalized to relative abundance and expressed as the percentage of one OTU with respect to the total number of OTU counts in each sample. For 16S data, statistical analyses were performed at the genus level for all samples, due to the lack of resolution power of the 16S V3–V4 region to enable accurate classification of all samples at the species level. Statistical comparisons were not established among ITS1 samples due to the reduced number of swabs with fungal presence (N = 13).

### 2.4. Statistical Analysis

Unless otherwise stated, all *p*-value cut-offs were set to 0.05. To determine whether lubricant application had any significant effect on the vaginal bacteriome, a permutational multivariate analysis of variance (PERMANOVA) was implemented using the *adonis2()* function from “Vegan” version 2.6-4 due to absence of normality, as revealed by a Shapiro–Wilk test. PERMANOVA tests for differences between group centroids and assumes that variances are homogeneous between groups [23,24]. To ensure that any differences between lubricant groups or timepoints were not induced by differences in variances (PERMANOVA assumption), a multivariate dispersion test (two-sided with a post-hoc Tukey’s test) with Bray–Curtis dissimilarity measures was conducted prior to PERMANOVA using the *betadisper*() and *permutest()* functions from “Vegan”. The beta-dispersion test showed a significant *p*-value (*p* < 0.05) between lubricant groups but not between timepoints within lubricant groups. Therefore, the PERMANOVA test was conducted individually for each lubricant group as the variances between lubricant groups were significantly different, hence PERMANOVA assumptions were not met between lubricant groups. Moreover, the permutations of the PERMANOVA test were restricted to occur within subjects only by using the *how()* and *Within()* functions from the package “Permute” version 0.9-7, to ensure data are not exchangeable between subjects in a repeated-measure study. To confirm that individual taxa at the genus level did not change significantly post-lubricant application, relative abundance changes from baseline were calculated and plotted as a function of timepoints. Significant differences were determined using a generalized linear mixed model (binomial error family, two-sided with a post-hoc Tukey’s test) with relative abundance proportions as the dependent variable, timepoints as the independent variable (and fixed effects), and subjects as random effects using the “glmmTMB” package version 1.1.9.

The “Vegan” package was also implemented to determine the bacteriome diversity between and within lubricant groups. To this end, alpha and beta diversities were calculated using measures of Shannon entropy and Bray–Curtis distance, respectively. The Shannon entropy index provides more inference about the community composition, placing greater emphasis on relative abundance of all taxa. The Bray–Curtis distance examines the abundances of shared and unique microbes between samples. Shannon entropy was illustrated in a long-format bar plot across timepoints for each lubricant group, and between age groups and menopausal stage at baseline only. Significant differences between timepoints, age groups, or menopausal stages were determined with the Kruskal–Wallis rank-based nonparametric test for non-normally distributed alpha-diversity data. Bray–Curtis dissimilarity measures were visualized in a non-metric multidimensional scaling (NMDS) ordination plot following an analysis of similarities test. Environmental variables with significant correlation (*p* < 0.05) were fit onto the NMDS ordination plots using the function *envfit()* from “Vegan”.

Differential analysis of bacteriome abundance at baseline between age groups was conducted with default parameters using the function *limma_voom()* available in the package “microbiomeMarker” version 1.6. Additionally, a series of generalized linear mixed models (two-sided with a post-hoc Tukey’s test) with relative abundance proportions as the dependent variable, additional measurements (i.e., pH, BMI, age, or vaginal humidity) as the independent variable (fixed effect), and subjects as random effects were fit by the package “glmmTMB” using a binomial error family to characterize if microbiome diversity and richness could be explained by any of the variables measured. It is worth noting that relative abundance proportions from all treatments and timepoints were used for this binomial regression approach, as there was negligible effect of product application across timepoints (PERMANOVA, *p* > 0.05). Hence, all relative abundance data were used for an exploratory association with the additional variables measured.

## 3. Results

**Figure 1:**
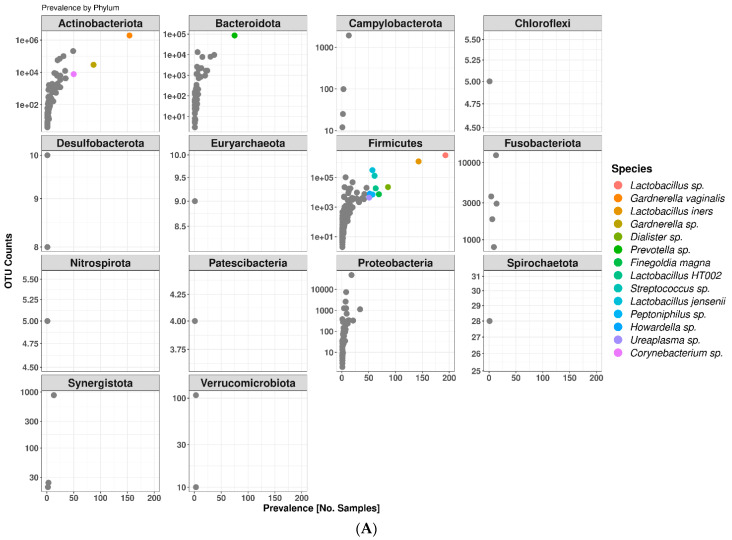

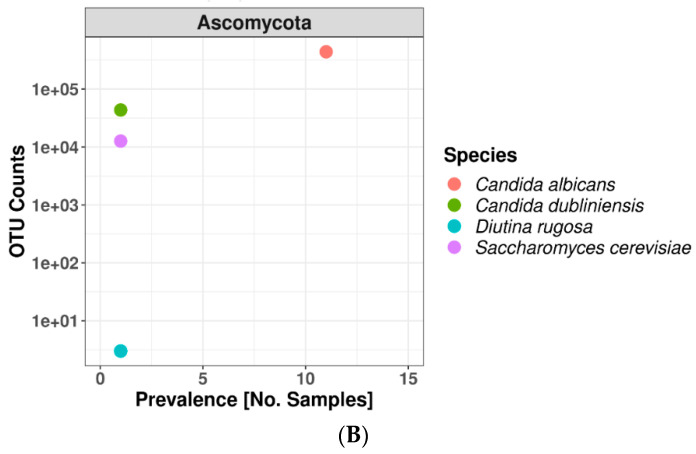
Bacterial taxa prevalence faceted by phylum in all 197 vaginal swabs (**A**) and fungal taxa prevalence grouped by Ascomycota phylum in 13 vaginal samples from 6 subjects (**B**). Prevalence is defined as the number of times a taxon is observed at least once in a sample (X axis). Colored bacterial circles represent species that were prevalent in at least 50 samples. Grey bacterial circles represent species that were prevalent in less than 50 samples.

The study subjects were colonized by a total of 14 bacterial phyla representing 230 genera and 415 species. Firmicutes was the most abundant phylum representing 65.8% of the classified OTUs (Figure 1A). Within this phylum, *Lactobacillus* were the dominant genus, accounting for 62.7%. The remaining 3.1% of the Firmicutes phylum were represented by *Streptococcus* (1.7%), *Megasphaera* (0.6%), and other genera with less than 0.5% representation. Actinobacteriota was the second-most represented phylum (31.3%) with the *Gardnerella*-dominated genus accounting for 25.5%, *Atopobium* for 2.8% (now known as *Fannyhessea* for the *vaginae* species), *Bifidobacterium* for 1.8%, and *Alloscardovia* for 1.3% of classified OTUs. The remaining genera within the Actinobacteriota phylum accounted for less than 0.5% of classified OTUs. Other phyla such as Bacteroidota (1.8%) and Proteobacteria (0.83%) were poorly represented but together with Firmicutes and Actinobacteriota accounted for 99.73% of classified OTUs. The remaining 10 phyla colonizing the study subjects represented less than 0.3% of the classified OTUs. 

Unlike bacteria, fungal colonization was poorly represented across subjects. Only 13 swabs sampled from 6 subjects showed presence of fungal taxa. Ascomycota was the only phylum detected with the *Candida*-dominated genus accounting for 97.5% of classified OTUs. *Candida albicans* represented the most prevalent fungal species which was identified in 11 vaginal swabs, accounting for 88.7% of classified fungal OTUs. The remaining fungal OTUs were classified as *Candida dubliniensis* (8.8%), *Saccharomyces cerevisiae* (2.6%), and *Diutina rugosa* (0.01%) (Figure 1B). It is not uncommon to find poor mycobiome representation in the human vagina of healthy individuals with *C. albicans* being a key player [25,26]. In addition, active yeast infection was one of the universal exclusion criteria of this clinical study, thus it was expected that not all healthy individuals would have detectable levels of vaginal mycobiome.

Given the limitation of partial 16S rRNA (V3–V4 hypervariable region) sequencing, and the fact that genomically-distinct species have similar 16S rRNA gene sequences [27], classification at the species level was only accomplished for taxa with exact matches between ASVs and sequenced reference strains using the *addSpecies()* function available in the “Dada2” package. At the species level, the most represented taxa in the study subjects were unclassified *Lactobacillus* spp. With a relative abundance mean of 39.8%, followed by *Gardnerella vaginalis* (24.3%), *Lactobacillus iners* (15.3%), *Lactobacillus jensenii* (3.9%), and *Atopobium vaginae* (now known as *Fannyhessea vaginae*, 2.6%) which collectively accounted for more than 80% of the vaginal bacteriome diversity.

**Figure 2:**
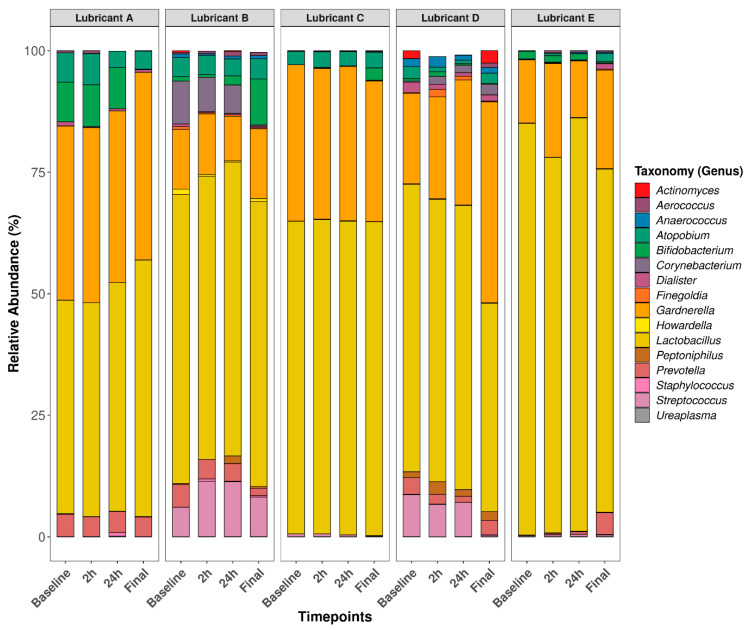
Relative abundance means of the most dominant bacterial genera as a function of timepoints, faceted by treatment groups. A within-subjects PERMANOVA test showed no significant differences between timepoints for each treatment group at *p* = 0.05 (Supplementary Table S5). *Ureaplasma* sp. (colored in gray) is difficult to distinguish in the plot as this taxon had less than 1.5% relative abundance for every sample, however it was consistent across timepoint thus it survived all filtering steps.

Poorly represented taxa, including those that were present fewer than three times in at least 20% of the samples, were removed for downstream visualization and statistical analyses yielding a total of 16 classified taxa. A beta-dispersion test was conducted between lubricant groups at baseline and between timepoints for each lubricant group, resulting in significant differences (*p* < 0.05) between lubricant groups only. This indicates that heterogeneity of variances was observed among lubricant groups, hence PERMANOVA assumptions were not met. Particularly, the baseline vaginal bacteriome variance from subjects enrolled into the lubricant E group differed significantly from subjects enrolled in lubricants A and B prior to lubricant application (Supplementary Table S5; Beta-dispersion test). Regardless of the beta-dispersion test outcome, it is worth noting that the goal of this study was not to compare bacteriome changes between lubricant groups but within lubricant groups across timepoints. Additionally, the beta-dispersion test was conducted to confirm whether lubricant groups should be considered in the PERMANOVA model. Therefore, lubricant effect across timepoints was assessed individually for each lubricant group via a within-subjects PERMANOVA test, hence avoiding permutation between subjects. As a result, no significant bacteriome differences at the genus level were observed between timepoints, for each lubricant group (*p* > 0.05). In addition, relative abundance changes from baseline were calculated for each individual bacterial taxon at the genus level and plotted as a function of timepoints. No significant changes from baseline were observed for each taxon post-lubricant application (*p* > 0.05), indicated by a generalized linear mixed model (GLMM) with binomial regression. Alpha-diversity measure (Shannon entropy) was categorized by lubricant type and timepoints without having significant changes (*p* > 0.05) between timepoints for each lubricant group, as revealed by a Kruskal–Wallis test (Figure S1). Taken together, lubricant use did not significantly alter the vaginal bacteriome at the genus level for up to 4 weeks post-lubricant application when measured by relative abundances and Shannon alpha diversity. Relative abundance of the most represented genera (Figure 2) and classified species (Figure S2) are illustrated in bar plots as a function of timepoints and faceted by treatment groups. Relative abundance changes from baseline are provided in Supplementary Figure S4.

**Figure 3:**
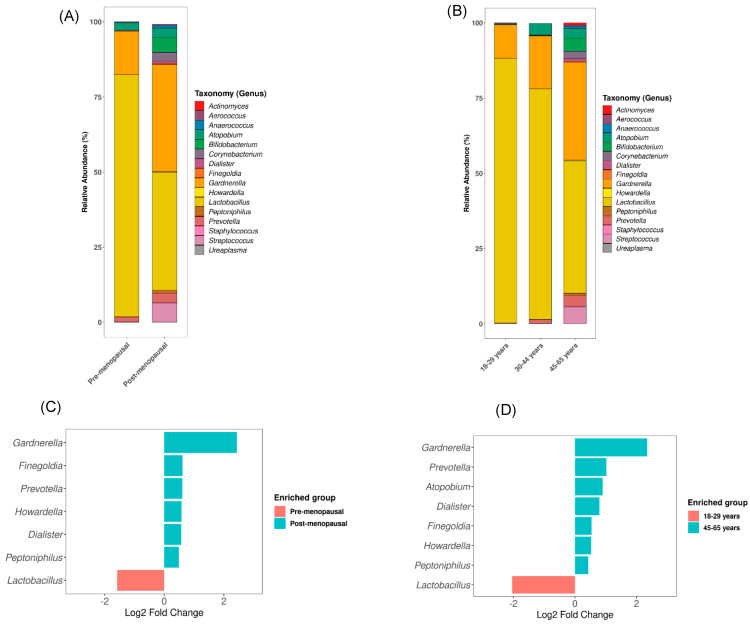
Relative vaginal bacteriome abundance (**A**,**B**) and differential enrichment analysis (**C**,**D**) of study subjects grouped by menopausal status (**A**,**C**) and age range (**B**,**D**) prior to lubricant application (baseline). For (**C**,**D**) panels, differential analysis of bacteriome abundance was conducted at the genus level with the function *limma_voom*, a *p*-value cut-off of 0.05 and log2 fold changes as follows: post-menopause vs. pre-menopause and 45–65 years vs. 18–29 years.

While lubricant use showed no significant genus-level effects on the vaginal bacteriome across all timepoints within treatment groups, the study subjects did show differences in bacteriome richness and diversity between groups with different menopausal status and ages. Post-menopausal subjects showed a significant increase of 6 out of 16 classified OTU genera accompanied by a significant reduction of *Lactobacillus* spp. (Figure 3A,C). These changes appear more gradual when the pre- and post-menopausal subjects were re-grouped by age range (Figure 3B). In this case, relative abundance changes observed for *Gardnerella* and *Lactobacillus* spp. can be gradually distinguished from younger subjects aged 18–29 years to those aged 45–65 years.

Differential enrichment analysis (DEA) revealed that *Lactobacillus* spp. were the only taxa with significantly higher relative abundance (*p* < 0.05) in pre-menopausal relative to post-menopausal subjects (Figure 3C). In contrast, post-menopausal subjects showed higher relative abundance of *Gardnerella*, *Finegoldia*, *Atopobium*, *Prevotella*, *Howardella*, *Dialister,* and *Peptoniphilus* than pre-menopausal subjects (Figure 3D). DEA among study subjects grouped by age confirmed that *Lactobacillus* enrichment was statistically significant in subjects aged 18–29 relative to 45–65 years old (*p* < 0.05). Conversely, *Gardnerella*, *Prevotella*, *Atopobium*, *Dialister*, *Finegoldia*, *Howardella* and *Peptoniphilus* significantly increased in 45–65-year-old groups relative to younger groups (*p* < 0.05). Interestingly, *Lactobacillus* represented the only significantly enriched genus in pre-menopausal subjects as well as in subjects aged 18–29 years. Conversely, *Gardnerella* was the most significantly enriched genus in post-menopausal subjects, including subjects aged 45–65 years. However, *Atopobium* was only enriched in subjects aged 45–65 years, but not in the post-menopausal group. This exclusive enrichment is likely due to shared microbiome communities between pre- and post-menopausal subjects that became more specific when subjects were re-grouped by age.

**Figure 4:**
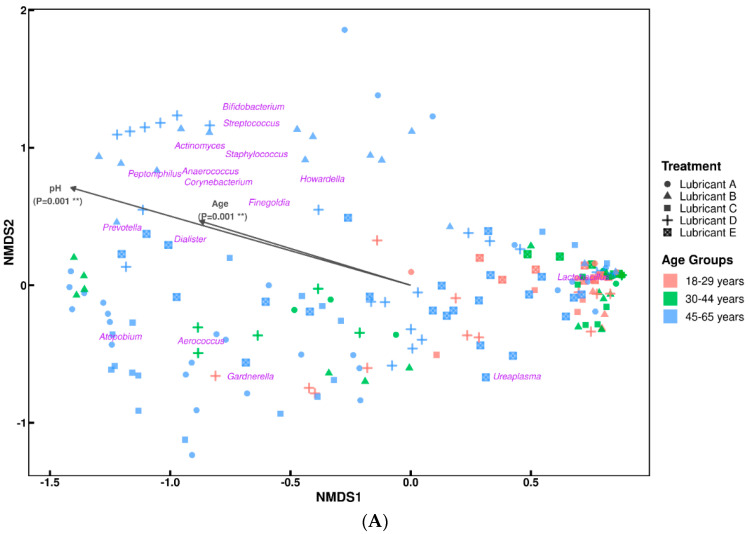

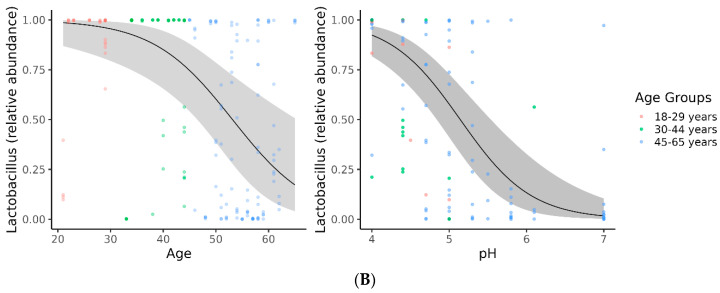
Global beta-diversity patterns of vaginal bacteriome communities (**A**) and a generalized linear mixed model with binomial regression of lactobacilli relative abundance (**B**). A: Beta-diversity patterns were grouped according to age ranges and treatment groups, and illustrated as NMDS ordination of the bacteriome composition dissimilarities (NMDS stress = 0.12). Environmental variables (age and pH, ** *p* = 0.001) are illustrated as plot vectors indicating strength and direction of the strongest correlations with bacteriome composition. BMI and vaginal relative humidity showed no correlation with bacteriome composition, therefore, the corresponding plot vectors are not shown in the plot. Bacterial genera appearing on the ordination represent the most abundant taxa for each age group. B: Generalized linear mixed model (binomial regression) visualization plot with relative abundance of lactobacilli as the dependent variable, pH and age as the independent variables (fixed effects), and subjects as random effects. Only pH and age were significantly associated with the relative abundance of *Lactobacillus* (*p* < 0.05). Gray shaded area represents the confidence intervals as per the analysis.

To assess whether overall species diversity (Shannon entropy) differed significantly between age groups and menopausal status, a Kruskal–Wallis test was implemented at the baseline. No changes in alpha diversity were observed between 18–29- and 30–44-year-old subjects, only between these two and the group aged 45–65 years with *p* < 0.05, indicating a higher bacterial diversity in the older population of female subjects (Figure S3). These differences in bacterial diversity were also observed when assessing the dissimilarities between the age group communities through beta-diversity measures (i.e., how microbial assemblages change along environmental gradients). The resulting NMDS ordination highlighted gradual bacterial community changes from groups aged 18–29 to 45–65 years with some overlap between groups (Figure 4A). This was further confirmed by an analysis of similarities test between age group communities (R = 0.2, *p* < 0.01), where the low R value indicates some dissimilarities between groups with high overlap of bacterial composition.

Additional data and measurements collected during the study (i.e., pH, relative humidity, age, BMI) were correlated with the beta-diversity measures (Figure 4A). However, only pH and age variables showed a significant correlation with the ordination plot. It is unclear if changes to the vaginal bacterial composition lead to pH changes, or if pH changes precede an overall change to the vaginal microbiome. Overall, the NMDS analysis indicates that *Lactobacillus* was commonly present across all age groups, however, with increasing vaginal pH and age, lactobacilli decreased while other bacterial taxa were detected.

Further exploratory analysis of associations between relative abundance and measures of pH, BMI, age, or vaginal humidity revealed that only pH and age were significantly associated with *Lactobacillus* relative abundance (Figure 4B). The remaining taxa showed no significant associations with the additional variables measured. As revealed by the analysis, an increase in vaginal pH from 4 to 7 was typically associated with decreased lactobacilli populations in the vaginal bacteriome of the study subjects. The analysis also revealed that lactobacilli populations tend to decrease as the subjects age. Taken together, pH and age factors appear to have a significant influence on vaginal lactobacilli populations.

## 4. Discussion

### 4.1. Main Findings

This randomized clinical trial showed that vaginal application of five water-based lubricants (four with an osmolality ≤ 1200 mOsm/kg and one with an osmolality of 5136 mOsm/kg) had no significant impact on vaginal bacteriome composition at the genus level for up to 4 weeks during lubricant application when measured by relative abundance proportions and alpha-diversity indexes. This finding was confirmed by a within-subjects PERMANOVA test with relative abundance as the independent variable, a Kruskal–Wallis test with alpha-diversity indexes as the independent variable, and individually for each taxon via a generalized linear mixed model (binomial regression) with relative abundance as the independent variable. However, vaginal microbiome communities differed significantly when the study subjects were re-grouped by age and menopausal status at the baseline. In particular, pre-menopausal women including those aged 18–29 years showed higher representation of vaginal lactobacilli than post-menopausal women, whose vaginal microenvironment was primarily colonized by *Gardnerella vaginalis*. Differential enrichment analysis highlighted significant differences between these two taxa which is in line with previous findings that *Lactobacillus* abundance decreases in post-menopausal women [6]. The present study revealed that vaginal bacteriome communities were distinct between older and younger participants. Specifically, younger females aged 18–29 years were predominantly colonized by lactobacilli. With increasing age, facultative or anaerobic bacteria such as *Gardnerella*, *Prevotella,* and *Streptococcus* gradually increased in abundance, while lactobacilli populations declined. This significant shift in vaginal bacteriome was strongly associated with pH changes (*p* < 0.05); from acidic conditions (pH 4–5) in younger age groups with predominant lactobacilli populations, to nearly neutral conditions (pH 6–7) in older females aged 45–65, where facultative and obligate anaerobes were most abundant.

### 4.2. Strengths and Limitations

The major strengths of this clinical trial included the rigorous experimental design and statistical planning, followed by extensive inclusion and exclusion criteria to select an approximate 50:50 ratio of pre- and post-menopausal women aged 18 to 65 years, which mitigated the risk of unbiased results. The study also benefited from the fact that 96% of eligible subjects were able to complete the tolerance and treatment phases without adverse effects, indicative of good tolerability of lubricants tested. Training given by clinical site personnel to eligible subjects demonstrated the amount of lubricant considered a single application and how to apply it to the vagina and intimate area. This strengthened the clinical trial measurements by standardizing the application method during the unsupervised treatment phase. It is acknowledged that this study had some limitations which could have been considered. Firstly, the number of pre- and post-menopausal subjects (N = 10) randomized to each lubricant treatment group were limited for the exploratory microbiome analysis. A higher number of eligible subjects would have enabled a more thorough examination of this exploratory endpoint to ensure lubricant groups have a similar distribution of vaginal microbiomes prior to lubricant exposure. Sample size was determined to achieve the statistical power for the primary endpoint (≥4-point increase in FSFI score from baseline to four weeks post-lubricant use) [17]. The majority of enrolled subjects (>90%) in this study were Caucasian, which may contribute to bias in microbiome profiling. Inclusion of diverse subjects would allow profiling of more comprehensive microbiome communities and therefore, improve understanding of lubricant effects in various parameters. As per the inclusion criteria, all participants suffered mild-to-moderate vaginal dryness; this may have impacted the collective generalizability of the results by assessing only a subset of the wider population. Additionally, a larger longitudinal study beyond 4 weeks could be key to understanding any potential long-term effect of vaginal personal lubricants. Microbiome profiling using a whole genome sequencing approach could have yielded more in-depth classifications at the species (and strain) level, and provided an opportunity for potential functional analysis. The study design could have benefited from the inclusion of a no treatment control group across timepoints; this would ensure that any changes in vaginal microbiome profiles due to internal or external factors other than lubricant use are considered. Lastly, using only the viable microbiome communities, which could have been confirmed by reducing extracellular DNA with endonuclease digestion prior to sequencing, would have optimized microbiome classification.

### 4.3. Interpretation

Overall, this repeated-measure profiling of the vaginal bacteriome conducted in this clinical study suggests that the personal lubricants tested have no significant impact on vaginal bacteriome communities for up to 4 weeks post-lubricant application. This finding differed from previous in vitro studies, showing that some vaginal lubricants appeared to inhibit growth of relevant vaginal taxa, in particular that of *Lactobacillus* spp. [11,14,15]. The antimicrobial effects of some commercial personal lubricants could be associated with the inclusion of ingredients such as broad-spectrum preservatives [28]. In this study, lubricants were formulated to include preservatives with less impact on the vaginal microbiome. Consequently, the inhibitory effects observed in peer-reviewed in vitro studies [11,14,15] may be due to the presence of a higher concentration of strong antimicrobial preservatives. The effects of personal lubricants on the vaginal microbiome remain poorly understood in vivo. Most of the published literature concerns the impact of these products on the vaginal epithelial barrier and mucosal inflammation, or ability to alleviate vaginal dryness, rather than vaginal microbiome integrity [8,29,30,31]. Only one case-control clinical study has been published regarding self-collected vaginal swabs before and after self-reported lubricant use without controlling for lubricant type and amount used during condomless sexual intercourse [32]. Regardless of the uncontrolled critical variables of this study, the authors concluded that the vaginal microbiome had no significant changes when assessed by similarity scores, diversity indexes, and community state types transition analyses. However, when assessing the mean relative abundance for each classified taxon individually, it was noted that *Lactobacillus crispatus* relative abundance decreased in subjects that self-reported to have used an unknown lubricant type and amount during sexual intercourse. There could be many plausible explanations for this *L. crispatus* reduction, such as uncontrolled usage of lubricants that may contain antimicrobial preservatives, or biases caused by self-collection of vaginal swabs without presence of a gynecologist or a trained technician. To our understanding, the present study represents the first randomized controlled clinical trial assessing the impact of personal lubricants which meet WHO guidance on osmolality on the vaginal bacteriome ecosystem in vivo.

The vaginal bacteriome has been indicated to have a crucial role in protecting the host against infectious diseases [1,2]. One of the mechanisms to prevent infection is the maintenance of an acidic vaginal microenvironment (pH 3.5–4.5) via the production of lactic acid, in which members of the genus *Lactobacillus* play a pivotal role [3,5]. Microbiome profiling in pre-menopausal subjects showed lower alpha diversity with *Lactobacillus* as the predominant taxa, whereas post-menopausal subjects showed higher alpha diversity with significantly reduced lactobacilli populations, and increased abundance of *Gardnerella vaginalis*. Therefore, the outcome of this exploratory study is consistent with published data, which suggests that the menopause is characterized by an increase in vaginal pH, reduction of lactobacilli colonization, and overgrowth of several facultative and obligate anaerobes [5,6,7]. Non-disturbance of the vaginal bacteriome following personal lubricant usage highlights novel findings which could potentially enhance the safety profile of personal lubricants. These findings suggest that users may apply appropriately formatted non-hormonal remedies to reduce vaginal dryness and improve sexual intercourse, without compromising the delicate balance of their vaginal microbiome, a crucial factor for vaginal health.

## 5. Conclusions

The present study demonstrated that vaginal application of the study lubricants caused no significant changes in the vaginal bacteriome at the genus level of pre- and post-menopausal subjects from baseline to 4 weeks post-application. Specific bacterial genera were identified as potential indicators for pre- and post-menopausal stages. The study also highlighted the significant association of vaginal pH and subject age in bacteriome diversity; particularly the reduced relative abundance of vaginal lactobacilli as the vaginal pH and subject’s age increased. Additional large-scale in vivo studies with more varied populations should be conducted to expand the scientific knowledge of the vaginal microbiome and its delicate microbial environment upon exposure to various intimate products.

## Data Availability

The raw FASTQ data generated and analyzed in this study have been deposited to the NCBI Sequence Read Archive and can be accessed with the BioProject accession number PRJNA1057945. Subject demographics are available in Supplementary Tables S1 and S2. All statistical analyses are available in the Supplementary Table S5.

## Supplementary Materials

The following supporting information can be downloaded at: https://www.mdpi.com/article/10.3390/microorganisms12091917/s1, Figure S1: Alpha diversity measures were represented by Shannon entropy across timepoints in a summarized long format bar plot faceted by lubricant groups. Outliers of Shannon entropy are highlighted in green. Significant differences between timepoints for each treatment were determined by Kruskal-Wallis multiple comparison test. No significant differences were observed between timepoints within each treatment group (*p* >> 0.05); Figure S2: Relative abundance means of the most dominant bacterial species classified with 100% confidence as a function of timepoints faceted by treatment groups. Unclassified taxa at the species level are denoted by their genus rank and “sp” abbreviation, where “sp” refers to one or multiple species. Due to the lack of resolution power of 16s V3–V4 amplicon data resulting in many unclassified species, a within-subjects PERMANOVA test was not conducted at the species level; Figure S3: Shannon entropy of total bacteriome data illustrated by age groups and menopausal stage at baseline. Outliers of Shannon entropy are highlighted in green. Significant differences between menopausal stages and age groups were determined by Kruskal-Wallis multiple comparison test, * = *p* < 0.05; Figure S4: Relative abundance changes from baseline plotted as a function of timepoints, faceted by taxon name and colored by lubricant group. GLMM with binomial regression indicated no significant bacteriome differences between timepoints within lubricant groups for each taxon. Corresponding p-values for each contrast comparison are available in Supplementary Table S5; Table S1: Descriptive statistics for subject demographics; Table S2: Counts and percentages of race at screening; Table S3: Osmolality and pH specifications of tested lubricants; Table S4: Metrics for 16S and ITS1 amplicon reads generated by Illumina MiSeq system; Table S5: Spreadsheet containing the output of all statistical analysis conducted in the study (Excel File name: Supplementary Table S5).
