# Supplementary material for: The In Vivo Effect of Water-Based Lubricants on the Vaginal Microbiome of Women from Varying Age Groups: Exploratory Analysis of a Randomized Controlled Trial
Source: Microorganisms. 2024 Sep 20;12(9):1917. doi: 10.3390/microorganisms12091917 (PMC11434374; doi:10.3390/microorganisms12091917)
Supplement: Supplementary file 1 [file microorganisms-12-01917-s001.zip › Supplementary Materials.pdf]

Table S1. Descriptive statistics for subject demographics.

		SP (N=183)*							
		Descriptive Statistics						95% CI	
		n	Mean	Standard Deviation	Minimum	Median	Maximum	Lower limit	Upper limit
**Age [years]**									
**Lubricant**	A	36	46.417	10.843	22.00	50.500	63.00	42.700	50.100
	B	36	44.583	13.712	23.00	44.000	64.00	39.900	49.200
	C	38	46.237	12.211	22.00	50.500	65.00	42.200	50.300
	D	37	44.649	13.977	20.00	51.000	63.00	40.000	49.300
	E	36	46.444	13.334	19.00	51.500	65.00	41.900	51.000
**Height [cm]**									
**Lubricant**	A	36	168.417	5.593	158.00	168.000	178.00	166.500	170.300
	B	36	167.500	6.345	153.00	168.000	180.00	165.400	169.600
	C	38	167.526	6.550	154.00	168.000	178.00	165.400	169.700
	D	37	169.378	6.193	155.00	169.000	181.00	167.300	171.400
	E	36	167.361	6.621	157.00	166.500	186.00	165.100	169.600
**Weight [kg]**									
**Lubricant**	A	36	73.236	18.182	48.80	70.800	138.80	67.100	79.400
	B	36	78.872	21.403	48.70	73.000	149.90	71.600	86.100
	C	38	77.379	13.460	56.30	77.100	107.30	73.000	81.800
	D	37	82.086	26.199	50.10	74.700	167.50	73.400	90.800
	E	36	75.422	11.875	46.90	77.350	95.30	71.400	79.400
**BMI [kg/m^2]**									
**Lubricant**	A	36	25.798	6.093	17.29	23.520	43.81	23.700	27.900
	B	36	27.938	6.421	17.89	26.445	47.31	25.800	30.100
	C	38	27.542	4.340	20.36	27.300	38.47	26.100	29.000
	D	37	28.542	8.777	17.54	25.940	56.62	25.600	31.500
	E	36	27.009	4.555	17.49	28.195	36.13	25.500	28.600

SP: Safety Population; CI: Confidence Interval.

**Data representative of full safety population (N=183), 50 subjects were selected at random (10 per treatment) as part of the microbiome analysis.*

Table S2. Counts and percentages of race at screening.

Race		SP (N=183)*									
		Caucasian		Asian		Black		Other		Total	
		n	(%)	n	(%)	n	(%)	n	(%)	n	(%)
Lubricant	A	36	(100.0)	0		0		0		36	(100.0)
	B	33	(91.7)	0		0		3	(8.3)	36	(100.0)
	C	35	(92.1)	0		0		3	(7.9)	38	(100.0)
	D	34	(91.9)	0		0		3	(8.1)	37	(100.0)
	E	34	(94.4)	1	(2.8)	0		1	(2.8)	36	(100.0)

SP: Safety Population.

**Data representative of full safety population (N=183), 50 subjects were selected at random (10 per treatment) as part of the microbiome analysis.*

Table S3. Osmolality and pH specifications of tested lubricants.

Lubricant	pH Range	pH Adjuster	Osmolality range (mOsm/kg)
**A**	5.1 - 5.8	Citric acid<br>Sodium hydroxide	5136
**B**	3.5 - 4.5	Sodium hydroxide	663 - 1063
**C**	3.5 - 4.5	Lactic acid<br>Potassium lactate	850 - 1200
**D**	3.5 - 4.5	Sodium hydroxide	780 - 1180
**E**	3.5 - 4.5	Sodium hydroxide	780 - 1180

Table S4. Metrics for 16S and ITS1 amplicon reads generated by Illumina MiSeq system.

Metrics	16S (N=197)		ITS1 (N=13)	
	Mean	Standard Deviation	Mean	Standard Deviation
**Raw Reads**	48,931	7,324	40,140	13,355
**Filtered Reads**	41,385	6,043	38,070	13,171
**Merged Reads**	38,734	6,574	38,044	13,170
**Recovery Percentage**	79	9	94	3

Table S5. Spreadsheet containing the output of all statistical analysis conducted in the study (Excel File name: Supplementary Table S5).

A)

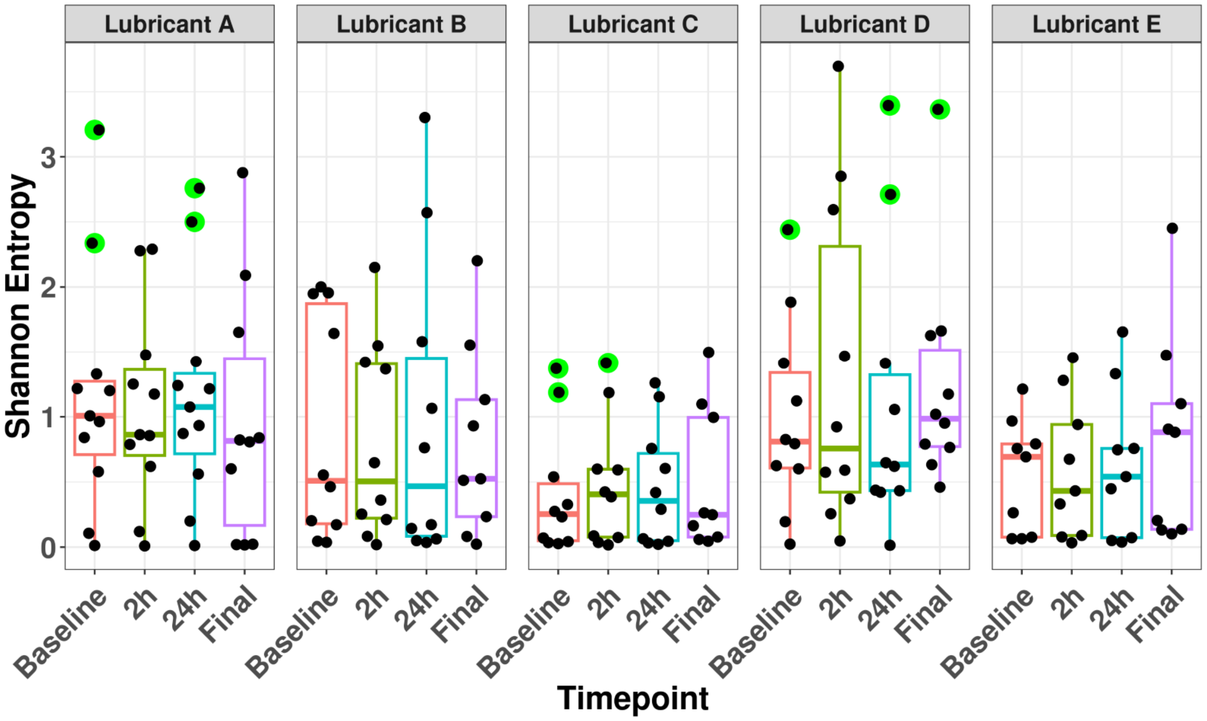

Figure S1. Alpha diversity measures were represented by Shannon entropy across timepoints in a summarized long format bar plot faceted by lubricant groups. Outliers of Shannon entropy are highlighted in green. Significant differences between timepoints for each treatment were determined by Kruskal-Wallis multiple comparison test. No significant differences were observed between timepoints within each treatment group (p>>0.05).

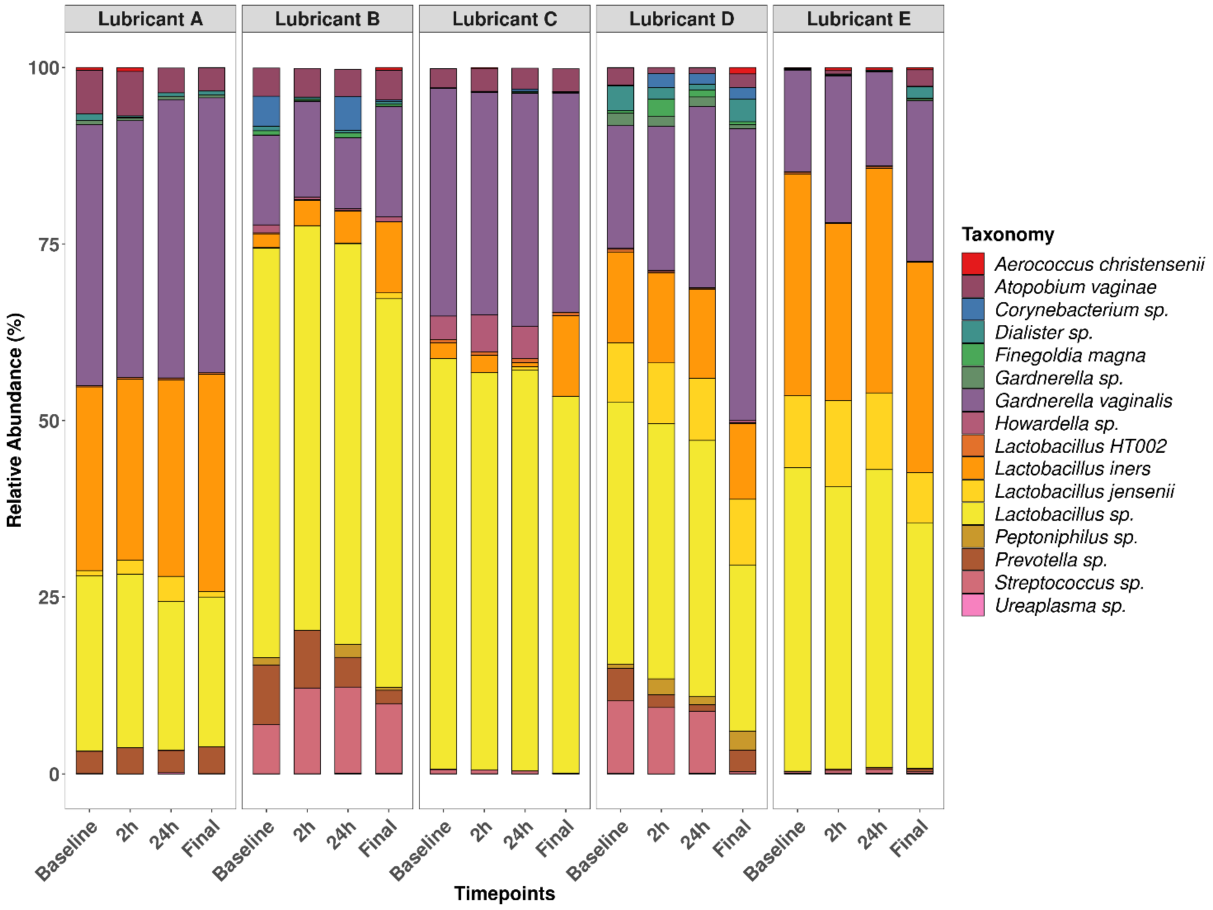

Figure S2. Relative abundance means of the most dominant bacterial species classified with 100% confidence as a function of timepoints faceted by treatment groups. Unclassified taxa at the species level are denoted by their genus rank and "*sp*" abbreviation, where "*sp*" refers to one or multiple species. Due to the lack of resolution power of 16s V3-V4 amplicon data resulting in many unclassified species, a within-subjects PERMANOVA test was not conducted at the species level.

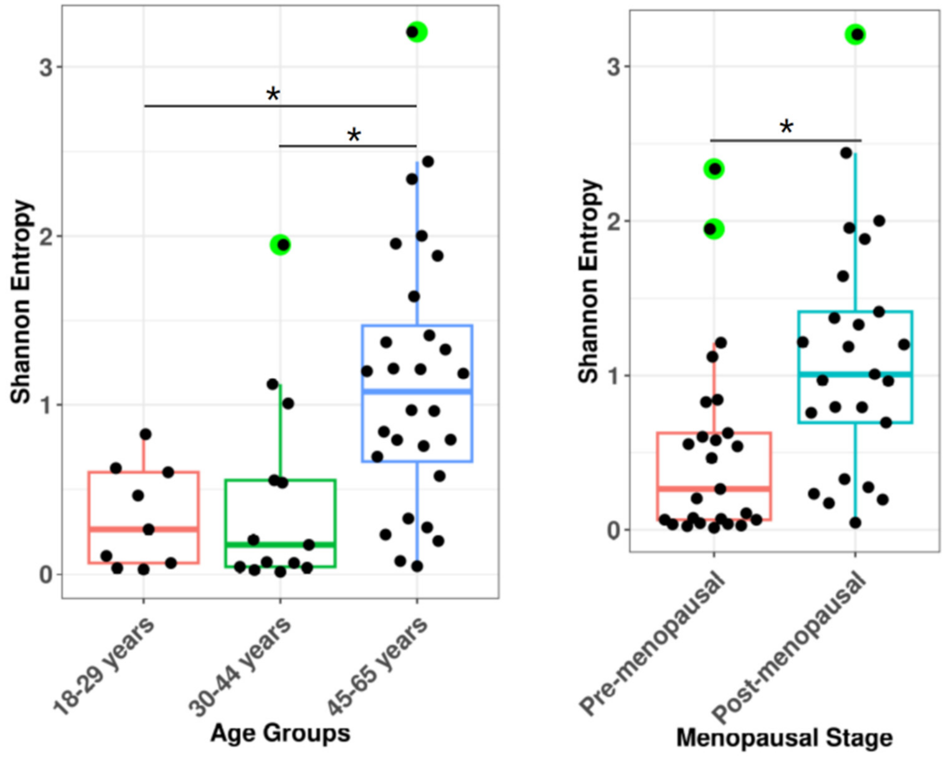

Figure S3. Shannon entropy of total bacteriome data illustrated by age groups and menopausal stage at baseline. Outliers of Shannon entropy are highlighted in green. Significant differences between menopausal stages and age groups were determined by Kruskal-Wallis multiple comparison test, * = p<0.05.

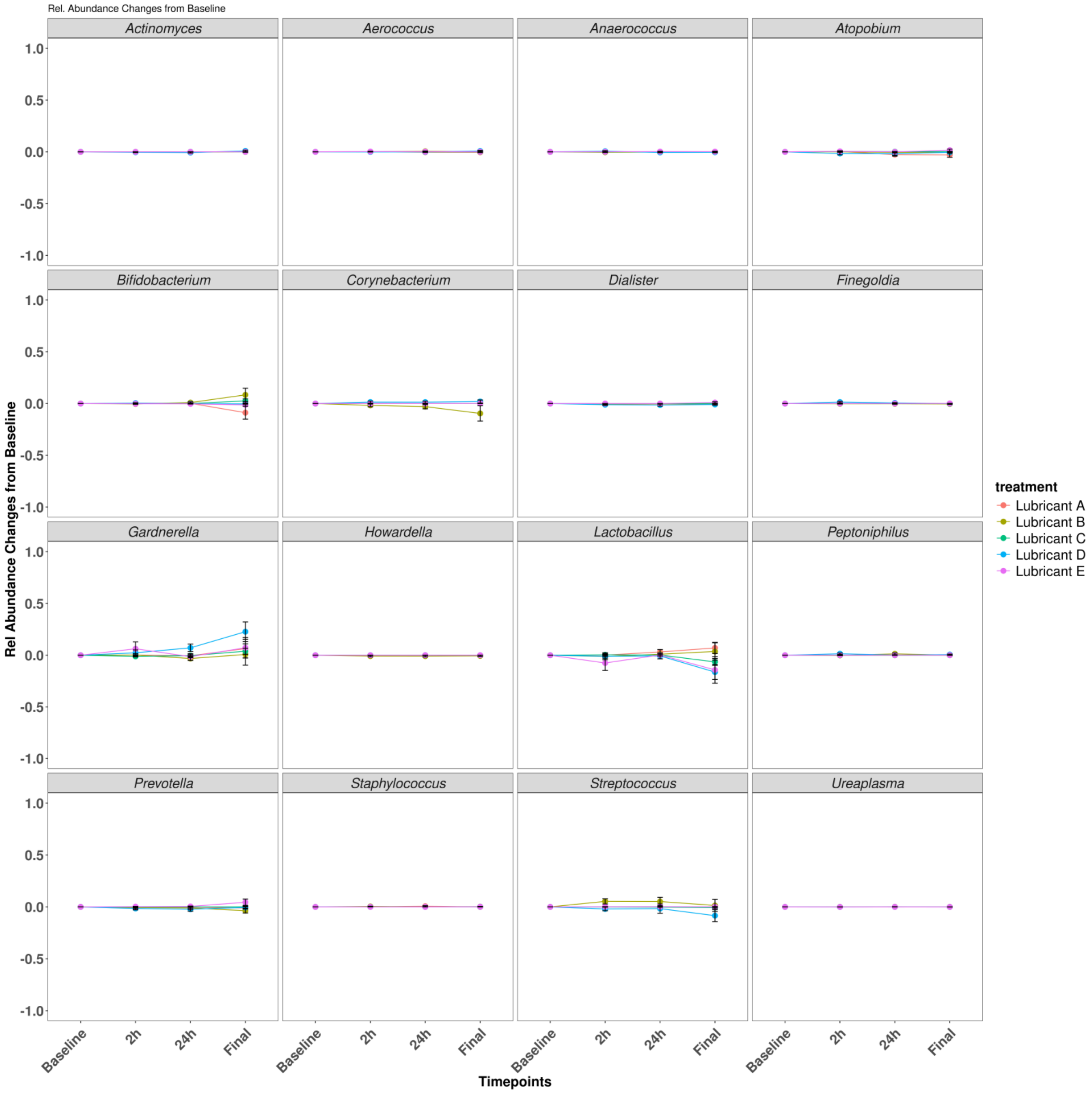

Figure S4. Relative abundance changes from baseline plotted as a function of timepoints, faceted by taxon name and colored by lubricant group. GLMM with binomial regression indicated no significant bacteriome differences between timepoints within lubricant groups for each taxon. Corresponding *p*-values for each contrast comparison are available in Supplementary Table S5.
